# Patient allocations in general practice in case of patients' preferences for gender of doctor and their unavailability

**DOI:** 10.1186/1756-0500-4-112

**Published:** 2011-04-08

**Authors:** Jostein Lillestøl, Jan Ubøe, Yngve Rønsen, Per Hjortdahl

**Affiliations:** 1 The Norwegian School of Economics, Bergen, Norway; 2 Institute of Health and Society, Medical faculty, University of Oslo, Norway

## Abstract

**Background:**

In some countries every citizen has the right to obtain a designated general practitioner. However, each individual may have preferences that cannot be fulfilled due to shortages of some kind. The questions raised in this paper are: To what extent can we expect that preferences are fulfilled when the patients "compete" for entry on the lists of practitioners? What changes can we expect under changing conditions? A particular issue explored in the paper is when the majority of women prefer a female doctor and there is a shortage of female doctors.

**Findings:**

The analysis is done on the macro level by the so called gravity model and on the micro level by recent theories of benefit efficient population behaviour, partly developed by two of the authors. A major finding is that the number of patients wanting a doctor of the underrepresented gender is less important than the strength of their preferences as determining factor for the benefit efficient allocation.

**Conclusions:**

We were able to generate valuable insights to the questions asked and to the dynamics of benefit efficient allocations. The approach is quite general and can be applied in a variety of contexts.

## Background

In some countries every citizen has the right to obtain a designated general practitioner. This is so in Norway, where a scheme, "fastlegeordningen", was initiated by law in 1999, see [1]. The scheme was implemented throughout the country by 2001, and some details on how it works may be found in Grytten and Sørensen [2]. Currently more than 99% of the population participate in the scheme. However, each individual may have preferences that cannot be fulfilled due to shortages of some kind. One example is the preference for having a general practitioner of the same gender, of the opposite gender, or be indifferent. It has been advocated that female patients have a stronger preference for doctor of the same gender than male patients. When female doctors are less frequent than male doctors, everybody cannot get a doctor according to their preference, unless there are unacceptable loads and unacceptable vacancies. To what extent can we expect that such preferences are fulfilled when the patients "compete" for entry on the lists? What changes in the distribution can we expect under changing conditions? In particular, in the case of many women with strong preference for a female doctor, how will the fraction of female patients assigned to female doctors expect to change when the fraction of available female doctors increases.

In Norway about 10% of the participants in the scheme change their general practitioner each year for some reason or other. We have no ambitions to describe individual actions at the micro level, e.g. how a patient having a doctor of the "wrong sex" initiates the search and may find a doctor of the "correct sex". A change will typically take place only at a vacancy at a doctor in the neighbourhood, and this varies not only between urban and rural areas, but also within certain regions. Initial assignment and decrement also complicates the matter, and it is probably futile to model a dynamic allocation process. A number of assumptions have to be made, that may be disputed and hard to verify empirically. Do we have alternatives that may provide insight to the dynamics and limitations of such a system?

## Methods

On the macro level it is well known that behaviour in many populations, among plants, animals and humans have cost minimizing traits. For humans this was formulated by Zipf [3]. Smith [4] gave a formal statement of the Zipf principle, saying that patterns with lower total costs are at least as likely as those with higher costs. On this basis Erlander and Smith [5] developed a general theory of efficient population behaviour, leading to a representation theorem for the feasible patterns. Among several special cases of this theory are situations leading to the so-called gravity model. Gravity type models are well known within in the urban geography literature as models for localization and interaction. The traces go back to Reilly [6] who studied retail relationships. More recent contributions to this theory are given by Jørnsten et.al [7]. Gravity models turned out to be quite robust under varied circumstances, and found applications in many fields, among them studies on travelling patterns and distribution of commodities in a network, both locally and in international trade. For early reviews on gravity and potential models for human interaction, see Carrothers [8] and Haynes and Fotheringham [9]. The gravity model has also found applications within health care, either from the point of view of needs (regardless of willingness to pay) or demand, see Connor et. al. [10]. Studies with a spatial dimension have either focused on potential availability or revealed availability in an area, see Shannon and Dever [11] and Gesler [12].

Our problem of distribution of patients among doctors may be formulated within the gravity model as well. The model has its limitations, and is not flexible enough to pick up interesting problems on the micro level. However, recent work by Jørnsten and Ubøe [13] has provided opportunities for including more characteristics and restrictions of different nature. This is required if we think of using such models for planning purposes at the micro level.

This paper contains what we believe is new material to statisticians and health professionals, with respect to modelling opportunities and qualitative results. The paper is in two parts, the first is on macro modelling based on the simple gravity model, and the second is on micro modelling based on recent theory reported in Ubøe and Lillestøl [14]. These models have intrinsic features that are not transparent from the general theoretically derived formulas, and the purpose of the paper is to bring forward some interesting dynamic aspects of the models. This is done by numerical calculations, with algorithms implemented in Excel and in Matlab. Although the problem came out of the Norwegian patient list system, the modelling approach and qualitative results have general applicability.

### The gravity model

We first explain the general idea of a gravity model by an example from another context, after which we explain the analogy to the patient list problem. Consider the travelling between residence and work, constituting the nodes in a network, where the distances between the nodes are given. The distances will influence the preferences for the locations, and therefore the travelling patterns between the nodes. Formalizing this we have a set *I *of "departure nodes" and a set *J *of "arrival nodes" and a distance function *d(i,j) *defined for all pairs *(i,j) *in *I × J*. The gravity model then writes the probability of "travelling" from *i *to *j *as

where *c *is a coefficient expressing the sensitivity to distance. Sufficient assumptions on the marginals of *P(i,j) *lead to a unique solution of the gravity equation in terms of the coefficients *a*_*i *_and *b*_*j*_. These coefficients may be calculated by an easily programmed and fast converging iterative process. In practice the restrictions may be derived from observed travelling patterns. In the general theory it is more convenient to talk in terms of costs rather than distance, and the theory allows different types of costs (in the wide sense), e.g. direct travelling cost and travelling time. The only difference is that this requires two constant terms in the exponent, each with a *c*-coefficient.

Early on this was just an attractive model to represent data, and criticized by economists for its lack of foundation in economic theory. Later it turned out that assumptions on "efficient population behaviour" lead to the conclusion that the travelling probabilities could be expressed this way. The expression can be obtained under different assumptions both within the classical utility paradigm of individual behaviour and by various probabilistic theories of choice behaviour, among them maximum entropy considerations. The classical microeconomic paradigm of utility-maximization subject to appropriate budget constraints is based on strong assumptions on individual choice behaviour, while the probabilistic theories may be based on weaker assumptions, see Sen and Smith [15] for a survey and references to an extensive literature on the topic. We limit ourselves to state that a possible behavioural interpretation is that the formula represents the maximal independent interactions possible under the restrictions of the system.

The analogy to the above for the match of doctors to patients, is that we have four types of patients, males and females preferring doctor of the same sex or not, i.e. four "departure nodes", while we have two "arrival nodes", male and female doctors. Formally we can write *I = {ff, fm, mf, mm}*, where the first letter is the gender of the patient and the second letter is the preference for gender of doctor. Furthermore *J = {F, M} *with letters representing the gender of the assigned doctor. The analogy to distance is the felt nuisance of being assigned contrary to preference. As distance function one may simply use *d(i,j) = 0 *or *1 *according to whether the second letter in the "departure node" corresponds to or deviates from "the arrival node" *j*, for example *d(mf,F) = 0 *while *d(mf,M) = 1*. We have then implicitly assumed that the nuisance of having a doctor of "unwanted sex" is the same for men as well as for women. By setting *d(ff,M) = 2 *rather than *1*, we have expressed that the women feel this nuisance stronger than men. However, this raises the question about realistic specification of the differences and the strengths of the preferences.

Our gravity model has two main application opportunities:

- gain insight to the dynamics of change under changing conditions

- establish current preferences by estimating the model parameters from data

This paper considers the former opportunity, by computational examples covering a sufficient parameter range to draw general qualitative conclusions not apparent from the gravity formula itself. We may then make comparisons under different assumptions and study what is most likely to affect changes in observed pattern, e.g. how the fraction of females assigned to female doctors changes as the fraction of female doctors increases, which is the expected scenario in many countries in the years to come. The qualitative results obtained, were partly a surprise to the medical professionals involved in the project, and initiated some rethinking. The second opportunity is clearly also of interest. It turns out that a given allocation does not uniquely determine the distance structure, i.e. different preference structures may lead to the same allocation. This identifiability issue requires additional theory, and is the theme in Ubøe and Lillestøl [16], where available Norwegian data is used for illustration. We briefly return to this aspect in the closing section of the paper.

## Results

### Macro analysis: The gravity model

Throughout our analysis we assume a population with equal number of male and female patients. In order to expose the effects of potentially different preference structures for female and male patients, we start with a situation where 70% of the female patients would like a female doctor the most, if available, while 30% of them would like a male doctor the most. Among the male patients we assume an even distribution of 50% for male doctor and 50% for female doctor. In the gravity model we specify the "distance" equal to 0 for allocations in concordance with the preferences and "distance" equal to 1 for discordance between preference and allocation. We will then change the percentages and the distances so that their effects become clearly exposed. For this purpose we keep the even distribution of preferences among the males fixed. Even if we say that a certain fraction of patients would like to have doctor of a certain gender, the degree of nuisance by contrary allocation varies, both among the sexes and absolutely. Possible larger nuisance among the females than males, of having a doctor of the opposite gender contrary to preference, may be adjusted by enlarging the distance for this female group. The strength of nuisance in general may be adjusted by a suitable weighting of the distance structure, i.e. by the factor c in the gravity formula.

First we will look at how the fractions of females vary among doctors of each gender for increasing fraction of female doctors. They are given in Table 1 for preference strength c = 1.

**Table 1:** Fraction of female patients among female and male doctors

Fraction F-doctors	10%	20%	30%	40%	50%	60%	70%	80%	90%
Fraction f among F	0.564	0.561	0.557	0.552	0.546	0.538	0.529	0.519	0.509

Fraction f among M	0.493	0.485	0.476	0.465	0.454	0.443	0.433	0.424	0.416

Fraction of female patients among female and male doctors for different fractions of available female doctors. The case of preference for female doctors 70% among female patients and 50% among male patients and preference strength c = 1.

### 

We see that when the fraction of female doctors increases, doctors of both gender will have a smaller fraction of female patients. With few female doctors, the fraction of female patients among them will be approximately 56%, and only decrease slowly until there is a more even number of doctors of the two genders, thereafter the fraction of female patients decreases more rapidly towards 50% of female patients among them. With few female doctors we see that the fraction of female patients among the male doctors is slightly below 50% and have a similar decreasing pattern. We also see that if the fractions of female and male doctors are equal, the fraction of female patients among the female and male doctors will be 54.6% and 45.4% respectively.

**Figure 1:**
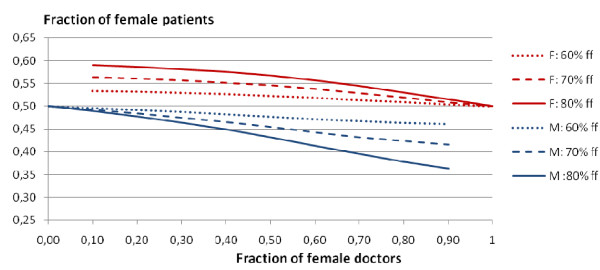
**Fraction of female patients**. Fraction of female patients as function of the fraction of female doctors, taken among female doctors (upper curves) and male doctors (lower curves), for 60% (dotted line), 70% (dashed line), 80% (solid line) of female patients preferring female doctors, and 50%-50% male preferences, all with preference strength c = 1.

Figure 1 shows for moderate weights (*c *= 1) the fraction of female patients among female and male doctors respectively as function of the fraction of female doctors in three situations, respectively where 60%, 70% and 80% of the female patients prefer female doctors and male patient preferences in all three situations are 50-50. The upper bundle of curves is for female doctors, and the lower bundle is for male doctors. In both bundles of curves the middle one corresponds to the numbers in Table 1. We see that all curves are monotonically decreasing, and that the decrease is slow until the fraction of female doctors is slightly above 50%, thereafter the decrease is more pronounced.

Similar computations for median/heavy weights (*c *= 2) for the same distance structure give a graph with the two bundles of curves further apart, but the difference is surprisingly small. Heavier preference weights (*c *= 3) gives results that do not deviate much from *c *= 2, and less so for small fraction of female doctors, where the fraction of female patients among female doctors is just increased from 56% to 58%, and eventually starts to decrease more rapidly above 50% female doctors. It may come as a surprise that the changes towards a larger fraction of female patients among female doctors are moderate as the preference strength increases. This may be interpreted as "limits to change" in a system where the felt nuisance for mismatch is the same for both gender, even if there is a strong majority of females feeling nuisance of a mismatch. However, if we change the preference structure itself, we may obtain large differences. We will return to this later.

Before turning to our next question, we note that the fractions considered above are tied together by a simple formula: Let *FF *and *FM *be the fraction of female patients on the list of female and male doctors respectively, and let *h *be the fraction of female doctors. The specification of *h*, *c (weight) *and *d *(distance) determines *FF *and *FM*, and implicitly the corresponding fraction of male patients among the female and male doctors respectively as *1-FF *and *1-FM*. However, *FF *and *FM *are also tied together. In a population of equally many female and male patients we have that *h*∙*FF + (1-h)*∙*FM = 1/2*, and so *FM = (1/2 - h *∙ *FF)/(1-h)*.

**Figure 2:**
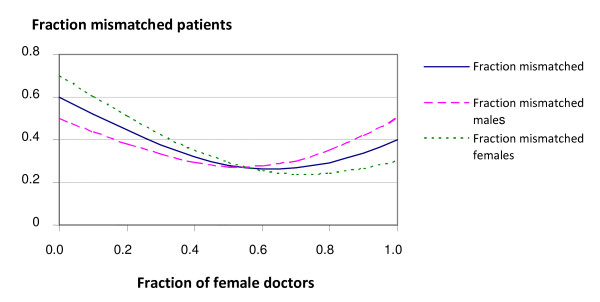
**Fraction mismatched patients as function of fraction female doctors**. Fraction mismatched patients as function of fraction female doctors in case of 70% of female patients preferring a female doctor and 50%-50% male patient preferences, (preference strength c = 1).

**Figure 3:**
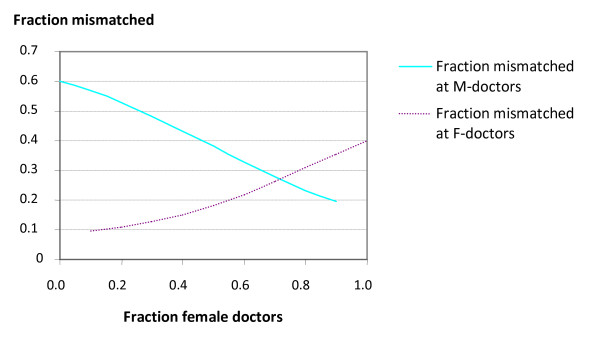
**Fraction mismatched at female and male doctors**. Fraction mismatched at female and male doctors in case of 70% of female patients preferring a female doctor and 50%-50% male patient preferences, (preference strength c = 1).

Next we will look at the fraction of patients not allocated according to preferences. For short, we name these patients mismatched. We may study the fraction of mismatched patients among each gender of doctors and the fraction of mismatched patients of each gender of patients as function of the fraction of female doctors. Computations using the gravity model for preference strength *c *= 1 gave the results in Table 2 and are illustrated in Figure 2 and 3.

**Table 2:** Fraction of mismatched patients

Fraction F-doctors	10%	20%	30%	40%	50%	60%	70%	80%	90%
Mismatched Total	0.519	0.443	0.376	0.320	0.281	0.262	0.266	0.293	0.339

Mismatched f-patients	0.601	0.508	0.423	0.349	0.290	0.251	0.234	0.239	0.263

Mismatched m-patients	0.436	0.378	0.329	0.292	0.271	0.272	0.298	0.347	0.416

Mismatched at F-doctor	0.094	0.108	0.127	0.150	0.181	0.218	0.261	0.308	0.355

Mismatched at M-doctor	0.566	0.527	0.483	0.433	0.381	0.327	0.277	0.233	0.197

Fraction of mismatched patients among each gender of patients and the fraction of mismatched patients at each gender of doctors for different fractions of available female doctors. The case of preference for female doctors 70% among female patients and 50% among male patients and preference strength c = 1.

### 

We see that the fraction of total mismatched is decreasing as the fraction of female doctors increases up to about the level where they are capable to accommodate the majority of female patients preferring a female doctor. Then the fraction of mismatched is increasing, since from then on more men are mismatched. A similar pattern is seen for both the female and male patients separately, except for the fact that the reversal for the males occurs at about 50%, as expected. Furthermore we see that the fraction mismatched at doctors of a given gender starts out low for the female doctors when they are few, and increases throughout as they become more abundant, ending with the situation where many of their patients are mismatched males. For male doctors the pattern is the opposite, starting out with a majority of male doctors with a large share of mismatched female patients, and ending up with few male doctors with mostly patients according to their preference.

**Figure 4:**
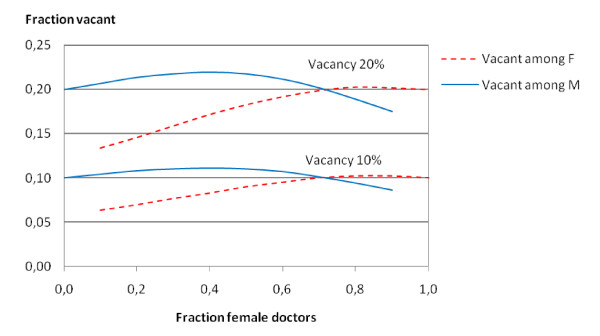
**Fraction of vacancies among doctors of each gender**. Fraction of vacancies among female doctors (dashed curves) and male doctors (solid curves) for total vacancy of 10% (lower curves) and 20% (upper curves) as function of the fraction of female doctors.

Our calculations on macro level are based on the assumption that the patient lists of all doctors are filled up, which means that adjustments to increase the total satisfaction at the micro level can be achieved by exchanging patients only. Some doctors will in practice of course have vacancies on their lists, and will be able to accept new patients according to their stated preference. It is not obvious how this should be implemented in the model. One possibility is to define a fifth fictitious patient category, representing an empty list position. If we are indifferent whether this happens to a male or a female doctor, we can represent this with a zero in the distance function. We have performed calculations according to this and with different fractions for total vacancy regardless of gender, and it turned out that the distribution of patient gender on the lists of both male and female doctors changed surprisingly little. However, the under-represented gender of doctors, in view of the preferences, will of course experience less vacancy on their lists. Figure 4 shows the fraction of vacancies for doctors of each gender for 10% and 20% total vacancy respectively for the situation above, where 70% of the female patients want a female doctor, and the male patients were distributed even.

If the take the situation with 30% female doctors and 10% total vacancy we have 7.6% vacancy among the female doctors and 11.0% vacancy among the male. With total vacancy of 20%, the numbers are respectively 15.8% and 21.8%. Not surprisingly, the fraction of vacancies for the two genders of doctors will be even when the fraction of female doctors is increased to about 70%, the fraction that matches preference among the female patients. When there is a lack of female doctors and the males get the larger fraction of vacancies, it may be of interest to introduce some regulatory measures so that the vacancies of both genders are about the same. In our model we may change the "distance" between the categories "empty list position" and female doctor from zero to a positive number. The magnitude of this number may be of interest to the regulators when balancing off conflicting objectives.

With vacancies, the quantities are tied together as follows: Let *t *be the fraction of total vacancies, and *TF *and *TM *be the fraction of empty list positions among female and male doctors respectively. Let as before *h *be the fraction of female doctors, and *FF *and *FM *be the fraction of female patients among female and male doctors respectively, but now taken to be among the non-vacant entries. The specification of *t*, *h*, *c *and *d *determines *TF*, *TM*, *FF *and *FM*, and implicitly the corresponding fractions of male patients among the female and male doctors as *1-FF *and *1-FM *respectively. *TF*, *TM*, *FF *and *FM *are, however, linked together since we in a population of equal number of men and women have that *h*∙*(1-TF) *∙ *FF + (1-h)*∙*(1-TM) *∙ *FM = ½*∙*(1-t)*. Given three of the quantities with capital letters, the forth is determined as well.

In the examples above we have assumed that more women (70%) than men (50%) feel some nuisance with a doctor of the opposite sex, while the felt nuisance is about equal for both genders. We have seen that this difference between the genders does not put appreciable pressure towards skew distribution of patient gender for the doctors. Only when this nuisance is felt stronger among women than men can we expect larger changes. If we again take the situation above, where 70% of the female patients will prefer a female doctor, and the male patients were distributed evenly, and where, as before, *d(mm, F) = 1*, but *d(ff,M) = 1*, *2*, and *3 *respectively, we get the results of Table 3 for the cases of 0%, 10% and 20% total vacancy and 30% female doctors (note that the fractions are with respect to the non-vacant entries)

**Table 3:** Fraction of female patients and vacancies for doctors of each gender

Fraction of vacancies	0%			10%			20%		
**Relative preference strength**	**1**	**2**	**3**	**1**	**2**	**3**	**1**	**2**	**3**

F-doctors: Fraction f-patients	0.557	0.684	0.794	0.557	0.679	0.782	0.556	0.675	0.772

F-doctors: Fraction vacant	0.000	0.000	0.000	0.069	0.048	0.031	0.130	0.094	0.064

M-doctors: Fraction f-patients	0.476	0.421	0.374	0.475	0.418	0.435	0.474	0.415	0.362

M-doctors: Fraction vacant	0.000	0.000	0.000	0.100	0.109	0.117	0.182	0.198	0.211

Fraction mismatched f-patients	0.422	0.325	0.245	0.417	0.312	0.223	0.412	0.301	0.207

Fraction of female patients and vacancies for doctors of each gender for 0%, 10% and 20% total vacancy for increasing nuisance of misplaced females, i.e. d(ff, M) = 1, 2, 3. The case of preference for female doctors 70% among female patients and 50% among male patients.

### 

We see how the increased relative preference strength between females and males forces the fraction of females among female doctors to increase, while it is reduced among the males. If we look at the case of 20% vacancies in the rightmost column of the table, we have 77.2% female patients among female doctors, and 36.2% female patients among male doctors. In both groups there are some who have fulfilled their primary wish, and some not, and the table also provides the fraction mismatched females patients under the varying circumstances. We have to increase the relative preference strength considerably in order for the fraction of females at female doctors to approach 100%. In fact, a raise of relative preference strength from 3 to 10 will give 97.6% females at female doctors, and just 0.4% misplaced female patients, everything else kept constant.

### Micro analysis: The extended gravity model

In the examples above we have studied the problem at the macro level, with a large and undefined number of patients and doctors, where the focus quantity is fractions. We have limited ourselves to situations with sufficient capacity to cover the demand for a doctor of some gender. In reality a limited number of doctors are available in the neighbourhood. Some of them may be fully booked, and we can imagine situations with waiting lists. We may also have additional characteristics separating both the patients and the doctors. Recent theory based on the idea of "efficient behaviour" offers an opportunity to analyze various situations on the micro level, see Jørnsten and Ubøe [13] for the general theory and Ubøe and Lillestøl [14] for theory in the current context. Here an extended gravity formula is derived under the assumption of "efficient system behaviour", assuming that for two allocations, the one with the highest total utility is more probable. See the appendix for the derived expression.

We will here give some general qualitative results coming out of the extended gravity formula, which is not transparent from the formula itself. Here we will mainly focus on the effect of differences and changes in the utility structure. As before we look at the four patient categories *mm*, *mf*, *fm*, *ff*, where the first letter denotes the gender of the patient and the second letter denotes the patient's preference for gender of doctor. Suppose that the categories mm and ff have moderate preferences for a doctor of the same gender, while fm and mf feel some, but not particularly strong nuisance by a doctor of the opposite gender. However, all categories feel a stronger nuisance by not being on the patient list of any doctor, i.e. being on a waiting list. For a start we assume that doctors are not penalized by not having their patient lists filled up. It is now more convenient to express the preferences in terms of "utility" (the negative of distance), taking both positive and negative values. A possible representation of the described situation by utility numbers is given in Table 4.

**Table 4:** Utility numbers for assignments

Group	mm-p	mf-p	Fm-p	ff-p	mm-w	mf-w	fm-w	ff-w	Vacancy
M-doctor	1	0	0	-1	-2	-2	-2	-2	0

F-doctor	-1	0	0	1	-2	-2	-2	-2	0


Utility numbers for assignments to doctor (as patient/waiting list) and vacancy.

### 

We will here consider situations with different number of available doctors of each gender. Assume first, to keep things simple, that all doctors have the same list length, and that we have the same number of patients in each of the four categories. The main findings are as follows: In the case of a deficit of doctors, the expected number of patients in each of the four patient categories will differ between male and female doctors, but be the same for all doctors of the same gender. However, the expected number and the distribution of patient categories on the waiting list will be the same for all doctors irrespective of gender. If we let the list lengths vary among doctors, but in a way so that the total numbers of patient entries are the same as above within each gender, it turns out that the fractions in each of the four patient categories are unchanged, while the number and distribution on the waiting list are unchanged. The relative distribution among the four patient categories on the patient list is therefore common for doctors of the same gender, so that the expected number is given by multiplication of the list length. Consider, as before, a situation with equal number of patients in each of the four patient groups, but so few that there is a deficit of patients. Now it turns out that all doctors of the same gender have the same share of vacancy on their lists, but different between the genders. Let us look at some specific examples, which exhibit some findings of general nature:

### Example

Given a population of 16 000 persons in an area served by 7 doctors, 4 male and 3 female, all with list lengths 2000, represented by the following vector with female numbers slanted (2000,2000,2000,2000,*2000,2000,2000*). With 4000 patients in each of the four patient categories, the expected distributions of the 16 000 patients on the 14 000 patient entries or the waiting lists are given in Table 5, for each of the male and female doctors (table sums deviating from the marginals are due to rounding errors):

**Table 5:** Expected distribution of patients on patient list and waiting list

Group	mm-p	mf-p	fm-p	ff-p	mm-w	mf-w	fm-w	ff-w	Vac.
M-doctor	835	509	509	147	51	84	84	67	1

F-doctor	102	458	458	981	51	84	84	67	1

Expected distribution of patients on patient list and waiting list for doctors of each gender (Doctors: 4 male, 3 female, Patients: 16 000, Patient entries: 14 000, equal list length of 2000).

### 

If we introduce varying list lengths (1000,2000,2000,3000,*1000,2000,3000*), i.e. the total number of entries for male and female doctors are as above, the number of patients for doctor 2, 3 and 6 are unchanged, while doctor 1 and 5 have cut their number of patients in half, and doctor 3 and 7 get their numbers multiplied by the factor 1.5. The waiting list numbers are the same for all seven doctors.

Now suppose we have 12 000 patients served by the 7 doctors, having 14 000 entries, but with varying list lengths as above, so that we have 14.3% total vacancy. Again assuming equal number of patients in the four categories, this time 3000, the model gives the allocation in Table 6, where we have lumped together the four waiting list groups, since they are all empty.

**Table 6:** Expected distribution of patients and vacancies (varying list length)

Group	mm-p	mf-p	fm-p	ff-p	Waiting	Vacancy
M-doctor 1 (1000)	343	223	223	62	0	149

M-doctor 2 (2000)	686	446	446	124	0	298

M-doctor 3 (2000)	686	446	446	124	0	298

M-doctor 4 (3000)	1029	669	669	186	0	447

F-doctor 1 (1000)	42	203	203	417	0	135

F-doctor 2 (2000)	84	405	405	834	0	271

F-doctor 3 (3000)	127	608	608	1251	0	406

Expected distribution of patients and vacancies for each of 7 doctors with varying list lengths (Patients: 12 000, Patient entries: 14 000, varying list length in parenthesis).

We see that all male doctors have 14.9% expected vacancy, while the female doctors have 13.5% expected vacancy.

### 

We are now ready to study what happens when the preferences are changed. First, if we change all disutilities by being on the waiting list from -2 to -3 (or even -5), there will be no change in the table, telling that the signal is picked up already at -2 for the case of plenty of vacancies and no loss for vacancy. If we introduce a loss for the doctor in case of vacancy, e.g. replace 0 by -1, we also get the same result as above. To create a difference, we must have a difference between genders with respect to felt loss. If the female doctors feel this stronger than the male doctors, they will have less vacancies. Some of this is of course fairly obvious, but shows that the model gives meaningful results throughout. For instance, if we change the losses from -1 to -2 for the female doctors, the expected vacancies among female doctors are reduced to 7.6%, while they among the male doctors are increased to 19.3.%. By taking -5 instead, the vacancies are changed to about 0.6% for female doctors and 24.6% for male doctors. One may ponder on the kind of administrative means required for such a transfer of welfare.

An interesting question is whether there are preference structures, where some doctors have vacancy, while there is a lack of doctors in the system as a whole. There are! One example is when the ff-group prefers to be on the waiting list of a female doctor, instead of being assigned to a male doctor.

In the discussion and examples for the micro-model, we have assumed an equal number of patients in the four patient categories. In our main macro-example of the preceding section we had 70% of the female patients favouring a doctor of the same gender, but 50% of the males. However, both models are general and accommodate any configuration. We can also extend the macro-model to include waiting lists.

In order to compare the results from the micro-model above with the macro-model, we may put heavy disutilities on waiting, so that this is ruled out. We will here consider cases where the number of entries exactly matches the number of patients, and put heavy disutility to wipe out vacancy as well. Consider therefore 14 000 patients to be assigned to 7 doctors, each with 2000 available entries totalling 14 000, and where 3 out of 7 doctors are female i.e. 42.8%. Assume first the same utilities for both gender, as we did for the macro-model (there measured by "distance"), and take the case of 70% of the female patients favouring a doctor of the same gender and 50% of the males. The distribution of the four categories *(mm, mf, fm, ff) *is (3500, 3500, 2100, 4900), and we take utilities -2 on waiting list and -2 for vacancy. We obtain the results given in Table 7.

**Table 7:** Expected distribution of patients (macro example comparison)

Group	mm-p	mf-p	fm-p	ff-p	Waiting	vacancy
M-doctors	725	348	435	487	5	5

F-doctors	198	700	119	980	5	4

Expected distribution of patients for each of 7 doctors with equal list lengths 2000 and patients in categories (mm, mf, fm, ff) = (3500, 3500, 2100, 4900), with disutilities -2 for each on a waiting list and each vacancy (for comparison with macro example).

### 

By changing the disutility for waiting and vacancy from -2 to -4 there will be none waiting and no vacancies. This gives the fraction of female patients assigned to male doctors 46.2% and to female doctors 55.1%, which is close to the numbers obtained for the macro-model.

On the other hand, if we consider the situation of stronger affection among the females mentioned above, we may specify the utilities as in Table 8.

**Table 8:** Utilities to reflect stronger affection and dislike

Group	mm-p	mf-p	fm-p	ff-p	mm-w	mf-w	fm-w	ff-w	vacancy
M-doctor	1	0	0	-2	-4	-4	-4	-4	-4

F-doctor	-1	0	0	2	-4	-4	-4	-4	-4

Utilities to reflect stronger affection among females and stronger dislike of vacancy and patients on waiting list.

### 

The results for the micro-model and the macro-model coincides, and the computation gave the results in Table 9, for some group fractions of females preferring female doctors.

**Table 9:** Fraction of female patients among male and female doctors

% ff vs fm	10-90	20-80	30-70	40-60	50-50
f among M-doctors	0.303	0.273	0.240	0.206	0.180
f among F-doctors	0.763	0.802	0.846	0.890	0.923

Fraction of female patients among male and female doctors for different fractions of female patients preferences and stronger affection among female than males (for comparison with macro model)

### 

At this point we are reminded that we can obtain major differences for the macro-model as well. It all depends on differences in the preference structure of the genders, and not so much on the number in each category and the absolute preference strength. In order to illustrate the sensitivity to change we take a macro example (with no vacancies and no waiting list), with equal number of patients in the four patient categories and utility structure as in Table 10.

**Table 10:** Utility structure to explore the sensitivity to change

Group	mm-p	mf-p	fm-p	ff-p
M-doctor	1	0	0	-x

F-doctor	-1	0	0	x

Utility structure to explore the sensitivity to change in female preferences for female doctor, where x is a positive number, typically greater than one.

### 

**Figure 5:**
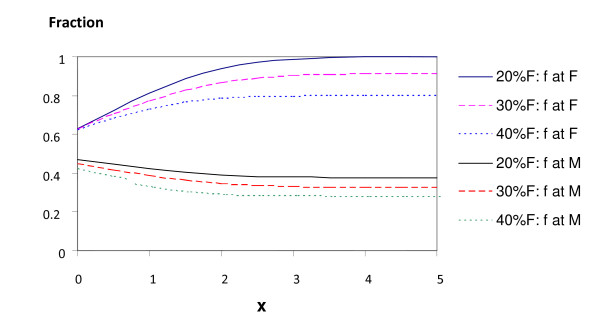
**Fraction of female patients among female and male doctors as function of disparate utility parameter**. Fraction of female patients among female doctors (upper bundle of curves) and male doctors (lower bundle of curves) as function of disparate utility parameter x for different fractions of female doctors 20% (solid line), 30% (dashed line) and 40% (dotted line).

In Figure 5 we plot, as function of x of Table 10, the fraction of female patients among female doctors (top three curves) and among male doctors (bottom three curves) for three fractions of female doctors 20%, 30% and 40% (in this order from top). Equal preferences correspond to x = 1, and the most relevant part of the curves is to the right of this. We see that the disparity of utility affects the female doctors more than the male doctors in this region, and that there is not much change beyond x = 3.

## Discussion

The theory and examples above aimed at giving some insights to qualitative dynamic issues, e.g. what affects the level and changes of allocations in a system competing for resources. We have tried to do this by means of models representing benefit efficient behaviour, and analyzing the issue related to a specific context, that of patients having preferences for the gender of their assigned doctor. One major finding here is that the number of patients wanting a doctor of the underrepresented gender is less important than the strength of their preferences as determining factor for the benefit efficient allocation. Qualitative insights of this kind may be of value to decision makers at the general policy level. For applications in the more local setting, in a specific country or region within a country, we need the statistics of the current situation with respect to the availability of doctors and the strength of preferences among the patients. This may be done by a suitably designed questionnaire, and may be a research challenge in itself. Another approach would be to use observed allocations, in combination with stated preference for gender, and from this infer the complete utility structure.

The Norwegian system by which every inhabitant has the opportunity of having a designated general practitioner was introduced in the year 2001 and is monitored by the authorities. Detailed information on availability of doctors and list composition and vacancies are available, and may serve as a laboratory for both research and applications. To give an idea of the kind of data available: At the end of the year 2004 the fraction of doctors with open lists was about 55%, 44% among female doctors and 59% among males in the country at large, while the corresponding numbers in the capital Oslo were 76%, 61% and 85%. Such numbers are available also regionally and locally. Movements over time are noticeable, and may indicate that the preference for a doctor of the same gender have increased since the system was introduced. However, part of this may be due to a tendency for most newborn to be assigned to the doctor of their mother. This raises an additional challenge, both for revealing real preferences and interpreting allocation data. It is possible that the solution to this is to combine the two approaches mentioned above.

We will here briefly address the so called inverse problem of inferring the utility structure from an observed allocation, leaving out the complications mentioned above. We are now facing a situation where different utility structures may lead to the same allocation, and consequently a given allocation will not uniquely determine the utilities. Some extra assumptions have to be imposed in order to identify the preference structure uniquely. In technical terms, we have an identification problem, which was solved theoretically in Ubøe and Lillestøl [16]. Here the general theory is illustrated by data on gender of patients, preferred gender of doctor and assigned gender of doctor extracted from the official panel survey of Norwegian living conditions ("Levekårsundersøkelsen 2003"). This work revealed, not surprisingly, a structure where the felt nuisance of a mismatched male patient who wants a female doctor was less than the corresponding mismatch for female patients wanting a female doctor. Perhaps more surprisingly, for both male and female patients, the felt nuisance of getting a female doctor when wanting a male was considerably higher, and highest for female patients. However, the response rate on these specific questions in the survey were low (a finding of some interest in itself), and more reliable data on the issue will be welcomed. This may give the authorities some clues with respect to "where we are and where we may be headed", in the most likely scenario of increasing fraction of female doctors.

## Conclusions

Based on a novel approach to allocations we were able to gain some insights to the dynamics of assignments according to gender preferences for doctors in general practice, in case of gender scarcity to fulfill the preferences. In particular we got some answers to the questions asked by the practitioners relating to likely changes when the scarce resource (i.e. female doctors) is on the rise. The approach is quite general and can be applied in a variety of contexts involving allocations according to preferences, with restrictions on fulfillment.

## Competing interests

The authors declare that they have no competing interests.

## Authors' contributions

The first author JL is responsible for the details of this work, which is partly based on recent theories developed by him and the second author JU. The third and fourth author (YR and PH) described the context, posed the problem and participated in the exchange of ideas that were crucial for the work. All authors have read and approved the final manuscript.

## Appendix: The benefit efficient micro model

Assume *p *patient categories (named i = 1,2,...,p) and *q *doctors (named by *j = 1,2,...,q*) of *r *different types (named by *k = 1,2,...,r*). The situation in the main text, with patient categories being the gender combined with the preferred gender of the doctor, corresponds to p = 4 and r = 2. Each doctor has a patient list with a given number of available entries and in addition a waiting list. To each doctor we associate *2p + 1 *categories, first the p patient categories for registered patients (named by *i = 1,2,...,p*), then the same *p *categories for the patients on the waiting list (named in the same order by *i = p+1, p+2,...,2p*), and finally the category *i = 2p + 1 *for registration of possible vacant entries. Let for *k = 1,2,...,r*

*u(i,k) *= Utility for patients of type *i *assigned to a doctor of type *k *(for *i = 1,2,...,p*)

*u(i,k) *= Utility for patients of type *i *on the waiting list of a doctor of type *k *(for *i = p + 1,...,2p*)

*u(i,k) *= (Dis)utility per vacant entry of a doctor of type *k *(for *i = 2p+1*)

An assignment of all the patients to doctors and waiting lists, as well as vacancies, is judged by their total utility obtained by adding utilities over all patients. Let *P(i,j,k) *denote the probability of a patient/vacancy of type *i (i = 1,2,...,2p+1*) belonging to doctor no. *j (j = 1,2,...,q*) who is of type *k*. The assumption of "efficient system behaviour" amounts to saying that for two allocations, the one with the higher total utility is more probable. From this assumption it follows that the allocation probabilities can be written on the following form, see Ubøe and Lillestøl [14]:

where the *a*'s and *b*'s are coefficients determined by the restrictions in the situation, among others the list length of each doctor, and typically also that it is an equal number of patients of each gender to be assigned. As for the gravity model *c *is the weight put on the differences in the assigned utilities. A constant added to all utility numbers have no effect, since this is absorbed in the multiplicative constants *a *and *b*. If we multiply all utilities with the same positive number, we get the same solution, since the *c*-coefficient becomes rescaled as well, while the products are the same. Thus *c *may be taken as so-called "numeraire". Note that the obtained representation is quite different from anything obtained by expected utility maximization.

Once we have specified *c *and the utilities *u(i,k) *we have an equation system that can be uniquely solved numerically, for instance by extensions of the Bregman balancing algorithm, see Bregman [17] and Jørnsten and Ubøe [13].
